# Our New York Letter

**Published:** 1887-10

**Authors:** 


### Correspondence.

### OUR NEW YORK LETTER.

THE FOOD OF WORKING MEN, AMERICAN AND EUROPEAN—WHAT SHALL BE THE COLOR OF OUR SUMMER CLOTHING ?—A NEW PLAN FOR A MORGUE—PROPOSED REMOVAL OF THE BLOOMINGDALE ASYLUM—RETURN OF MEDICAL TOURISTS—CELEBRATION OF THE OPENING OF THE NEW COLLEGE OF PHYSICIANS AND SURGEONS.

*To the Editors Atlanta Medical and Surgical Journal:*

At the last convention of American scientists, which was held at Columbia College, Prof. W. O. Atwater read a very interesting paper entitled "Food of Working Men and its Relation to Work Done." After illustrating by colored charts the composition of all our ordinary foods, he stated that a workingman doing moderately hard labor requires every day about one-fourth of a pound of proteine, one-fourth pound of fat and one pound of carbo-hydrates. Comparative diet statistics of the various people of Europe and America were read to prove that the average allowance of the Europeans is below this standard, while Americans exceed these requirements. But he believed that in America more food is bought than is actually required, and that a good deal of it is wasted by families eating more than is necessary. The poor people, he said, get less for their money than the rich, as the latter are generally more intelligent and economical in buying their food. He also considered that working people are especially difficult to please in regard to meats, and generally want the finest grades of flour, for which they are obliged to pay high prices, while, on the other hand, many wealthy people are satisfied with cheaper, but just as nourishing, foods. He said the average poor man displayed very little economy in managing his household affairs, and that the only true anti-poverty society is the society of toil, thrift and temperance.

The American working people, he believed, were also better housed and clothed than their foreign brethren, and, owing to their better food, are able to accomplish a much greater amount of work than the Europeans. As they possess superior intelligence, and have such great opportunities offered them, we ought justly, he thought, expect great excellence from American artisans in all trades calling for a display of skill.

There has been somewhat of a discussion among medical men this summer in New York on the subject of the color of wearing apparel and its relation to heat, or, in other words, whether dark or light clothing is the cooler in hot weather. Dr. Willard Parker believes that thin woolen should he worn next the skin and that the outside clothing should be thin and light-colored. He says dark colors attract the heat,while light shades repel it. About the same time that he expressed this opinion, Dr. Cyrus Edson stated that it was better to wear dark clothes in summer. He admitted that black colors absorb heat when exposed to sunlight, but said that the heat will pass out through the black materials very quickly, and consequently radiation from the body will occur more rapidly in a person wearing dark clothes. As further evidence of the correctness of these statements, he said that in the arctic regions the animals have white coats for the purpose of retaining their bodily heat better.

The following are some of the arguments, pro and con, which have been used in discussing this subject: The color of the Polar bear was disposed of by saying that his coat is white because the surrounding snow is the same color, and he is therefore better hidden from his enemies, and is better able to come upon his prey unnoticed. It has also been said that he lives in a location where there is very little sunlight, and consequently would not be affected from this source either way. The experiment has been alluded to of placing several pieces of different colored cloth on snow and exposing them to the sun's rays, when it will be noticed that the snow under the dark colors melts the most rapidly. Melloni's statement that color will only influence luminous heat, but has no effect on its absorption when non-luminous, has also been quoted to prove that the color of clothing only plays an important part when exposed to the sun's rays. Regarding the color of animals, it has been said that the arctic grouse becomes white in winter and heather-colored in summer, according to the place it inhabits. The black color of the negro has not been considered of any significance in this connection, and his ability to withstand severe heat has been explained by the excessive development of his sweat-glands. Many other arguments have been brought out on this subject, but the bulk of medical opinion seems to lean towards Dr. Parker's views.

Some needed improvements are to be made in our city dead-house, or "morgue," as it is called. This is a building located at 26th street and East River, near Bellevue Hospital, where the unknown dead are kept for a certain time in hopes that friends may come and identify them. For a long time the methods of preserving these bodies have been very unsatisfactory, and complaints have been numerous on this account from those who have been obliged to bury friends found here. Various changes in the features of the dead have resulted from the present plan of placing a number of bodies in an apartment without any method of preservation. As soon as the required alterations can be made, a new plan will be adopted. A large iron box will be constructed on both sides and across the rear of the inside of the building and divided into receptacles, three deep, for the accommodation of bodies. Each receptacle will be furnished with a separate door, having a glass window to it. The bodies are to lie upon perforated boards. A low temperature will be maintained throughout this whole series of compartments by a large quantity of ice placed above the iron box, and pipes going to the centre of each receptacle will furnish a stream of cold air for every corpse. The work, when completed, is expected to supply accommodation for two hundred bodies, and will cost about $2,000.

There has lately been considerable talk about the proposed removal of the old Bloomingdale Insane Asylum. The present grounds and buildings cover about 670 city lots in a portion of New York where constant improvements are in progress. It is the desire of numerous real estate men to run streets through the asylum grounds, and otherwise advance the value of this part of the city. This institution is owned and managed by the New York Hospital Society, and last year the ground and buildings were valued at from four to five million dollars. This society owns a great amount of real estate, and is estimated to be worth about fifteen million dollars. Among their possessions is a three hundred acre farm at White Plains, not far from New York, and this has been mentioned as a favorable site; but it is as yet undecided where the new Bloomingdale Asylum will be located.

Many of our physicians are returning to the city from their summer vacations, but as yet the medical societies have hardly gotten under way in their fall work, and at present the Medical Congress, at Washington, is occupying the attention of a large number of our eminent doctors.

The College of Physicians and Surgeons will celebrate the opening of their new college building on September 29th by a dinner at Delmonico's. The affair will be under the management of the alumni of the college, and promises to be a very enjoyable event. A. F.

*September, 1887.*

